# Successful mLearning Pilot in Senegal: Delivering Family Planning Refresher Training Using Interactive Voice Response and SMS

**DOI:** 10.9745/GHSP-D-14-00220

**Published:** 2015-06-02

**Authors:** Abdoulaye Diedhiou, Kate E Gilroy, Carie Muntifering Cox, Luke Duncan, Djimadoum Koumtingue, Sara Pacqué-Margolis, Alfredo Fort, Dykki Settle, Rebecca Bailey

**Affiliations:** ^a^​ IntraHealth International Senegal, Dakar, Senegal.; ^b^​ IntraHealth International, Capacity*Plus* project, Washington, DC, USA; ^c^​ St. Catherine University, St. Paul, Minnesota, USA; ^d^​ University Research Company, LLC, Bethesda, MD, USA

## Abstract

**Background:**

In-service training of health workers plays a pivotal role in improving service quality. However, it is often expensive and requires providers to leave their posts. We developed and assessed a prototype mLearning system that used interactive voice response (IVR) and text messaging on simple mobile phones to provide in-service training without interrupting health services. IVR allows trainees to respond to audio recordings using their telephone keypad.

**Methods:**

In 2013, the Capacity*Plus* project tested the mobile delivery of an 8-week refresher training course on management of contraceptive side effects and misconceptions to 20 public-sector nurses and midwives working in Mékhé and Tivaouane districts in the Thiès region of Senegal. The course used a spaced-education approach in which questions and detailed explanations are spaced and repeated over time. We assessed the feasibility through the system's administrative data, examined participants' experiences using an endline survey, and employed a pre- and post-test survey to assess changes in provider knowledge.

**Results:**

All participants completed the course within 9 weeks. The majority of participant prompts to interact with the mobile course were made outside normal working hours (median time, 5:16 pm); average call duration was about 13 minutes. Participants reported positive experiences: 60% liked the ability to determine the pace of the course and 55% liked the convenience. The largest criticism (35% of participants) was poor network reception, and 30% reported dropped IVR calls. Most (90%) participants thought they learned the same or more compared with a conventional course. Knowledge of contraceptive side effects increased significantly, from an average of 12.6/20 questions correct before training to 16.0/20 after, and remained significantly higher 10 months after the end of training than at baseline, at 14.8/20, without any further reinforcement.

**Conclusions:**

The mLearning system proved appropriate, feasible, and acceptable to trainees, and it was associated with sustained knowledge gains. IVR mLearning has potential to improve quality of care without disrupting routine service delivery. Monitoring and evaluation of larger-scale implementation could provide evidence of system effectiveness at scale.

## Abstract (precis)

Health workers’ knowledge of contraceptive side effects increased substantially. The mobile phone approach was convenient and flexible and did not disrupt routine service delivery. Clear limitations of the medium are participants can’t practice clinical skills or have interactive discussions. Also, some participants had trouble with network reception.

## Teaser

Health workers’ knowledge of contraceptive side effects increased substantially after the refresher training. The mobile phone approach was convenient and flexible and did not disrupt routine service delivery. Clear limitations of the medium are participants can’t practice clinical skills or have interactive discussions. Also, some participants had trouble with network reception.

## BACKGROUND

Strengthening training programs for health workers is pivotal to increasing the number of qualified providers and improving service quality. In-service training reinforces and updates health care providers’ knowledge but is often expensive and requires providers to leave their posts.^1^

In-service training reinforces and updates providers’ knowledge but is often expensive and disrupts services.

The global health field increasingly recognizes the need to expand continuing education of health professionals beyond the classroom setting.^1^^,^^2^ Spaced education is an approach to distance learning in which content is spaced out and repeated over time in the form of multiple choice questions and detailed explanations. The presentation and repetition of information over time (spacing) and the testing effects inherent in this approach have been found to increase retention of clinical knowledge^3^^–^^5^ and skills.^6^^,^^7^

The spaced-education approach, in which course content is spaced out and repeated over time, has been found to increase knowledge retention.

Health care programs and providers increasingly use mobile phones and devices to deliver health information, collect information, diagnose disease, and improve adherence to clinical protocols,^8^^–^^11^ as well as to improve health worker performance through the provision of information and training.^12^ These types of mHealth applications can provide and expand distance education opportunities in rural and remote settings, where health workers have less access to computers than their urban counterparts or may have less connectivity to the Internet yet have mobile phone connectivity.^12^ However, many current mHealth applications to train health workers require smart phones or digital tablets and Internet connectivity,^12^^–^^15^ which is typically more expensive. Likewise, many mHealth applications use Short Message Service (SMS) text systems to provide training, guidance, and updates,^16^^–^^18^ but messages must be kept simple due to the limited number of characters that can be sent.

Interactive voice response (IVR) is a technology that uses any type of phone to deliver information via audio recordings and that allows users to provide feedback through their telephone keypad by pressing a number key. Companies frequently use IVR to respond to customer service requests and questions in an automated way. The IVR technology has also been used in medicine for many years in highly industrialized countries.^19^^–^^21^ Health programs are increasingly using IVR in developing countries to reach and educate populations that have high penetration of mobile phone ownership but low levels of literacy and Internet connectivity.^22^^–^^25^ Using IVR systems to provide training and support to health workers is less common. One substantive example, however, is the Mobile Academy, implemented by BBC Media Action as part of the Ananya project in Bihar, India, which has used IVR technology to deliver training on maternal and child health to more than 27,000 remote community health workers.^26^

With interactive voice response technology, information can be delivered to any type of phone via audio recordings.

In response to calls to explore and assess more effective ways to deliver in-service training^1^ and support family planning health workers with mHealth applications,^27^ the Capacity*Plus* project, funded by the US Agency for International Development (USAID), developed, deployed, and assessed a prototype mLearning system, using a combination of simple IVR and SMS, to deliver refresher training on management of contraceptive side effects and misconceptions to health workers in Senegal.

## MLEARNING PILOT DESCRIPTION

### Participant Selection

Senegal’s contraceptive prevalence rate for modern methods remains low, at just over 16% in 2012–2013, and about one-third of married women have an unmet need for contraception,^28^ despite significant efforts to improve access to and demand for contraception. Through the Maternal, Neonatal, and Child Health/Family Planning/Malaria Project (2006–2011), funded by USAID, IntraHealth International trained providers in family planning counseling and provision of contraceptives, encouraged facilities to integrate mother-and-child health services with other reproductive health services, and engaged and educated communities through public campaigns.

To test the feasibility of using a simple mobile technology to deliver refresher family planning training to providers, we coordinated with the Senegal Ministry of Health to purposively select 20 nurses and midwives working in public health facilities in Mékhé and Tivaouane districts in Thiès region.

Three criteria guided the selection of providers:

- Informed consent to participate in the pilot project
- Participation in the initial family planning training course conducted by IntraHealth International in 2008/2009
- Possession of a simple mobile phone for the training course

We limited the sample size of participating providers due to financial considerations and to facilitate rapid resolution of any unforeseen technical and logistical issues in the field associated with the first-time application of the IVR mLearning training system.

### Course Content

We designed a refresher training course that covered the management of contraceptive side effects and misconceptions, based on initial training and in direct alignment with the Senegal family planning national training curriculum and protocol as well as international guidelines.^29^^–^^31^ The course content covered general contraceptive side effects and misconceptions as well as specific content related to intrauterine devices (IUDs), implants, oral contraceptive pills, emergency contraceptive pills, condoms, and the lactational amenorrhea method (LAM). Stakeholders in Senegal, including a representative from the Ministry of Health, reviewed and approved the training course. The course content was delivered using the spaced-education approach,^5^^,^^32^^,^^33^ with 17 multiple choice questions and 3 true/false questions along with accompanying detailed explanations spaced and repeated over time. The Box presents an example of a training question and explanation. (See supplementary materials for the full training course packet in both English and French.) The audio questions and explanations were recorded in French by a health professional from Dakar, Senegal.

**BOX. Sample Refresher Training Course Content: Management of Contraceptive Side Effects and Misconceptions**

**QUESTION**

Which of the following statements on side effects is important to tell a woman who has decided to have an IUD inserted?

1. She can expect to have heavy bleeding and severe abdominal pain during the first week after insertion, and ibuprofen can be taken to alleviate the discomfort.
2. She can expect to have severe headaches, especially in the first few days after insertion, and aspirin can be taken to alleviate the pain.
3. She can expect some cramping and mild pain, especially in the first few days after insertion, and ibuprofen can be taken to alleviate the discomfort. **[Correct answer.]**
4. She can expect to have fever, chills, and unusual vaginal discharge during the first week after insertion. This is normal and will go away with time.

DETAILED EXPLANATION*

Cramping and mild pain are common side effects experienced during the first few days after insertion by women who have an IUD. Heavy bleeding, severe abdominal pain, severe headaches, fever, chills, and unusual vaginal discharge are NOT common side effects and may in fact be signs of complications that require medical attention.

If a woman has an IUD inserted, reassure her that cramping and mild pain are common, especially in the first few days after insertion. Explain to her that cramping and mild pain are also common in the first 3 to 6 months, particularly during menses. These are not harmful and usually decrease over time.

To alleviate discomfort from cramping and mild pain, suggest that the woman take 200 to 400 milligrams of ibuprofen or take another pain killer. Do NOT offer her aspirin. Women who have an IUD often experience heavier menstrual bleeding and should not take aspirin, because it inhibits clotting and thus can increase bleeding.

If cramping continues or occurs outside of menstruation, evaluate for underlying health conditions, treat and/or refer the woman for treatment. If no underlying condition is found but cramping continues and client finds it unacceptable, discuss removing the IUD with the woman and switching methods. Discuss alternative methods with the woman.

*Learner hears this detailed explanation after selecting a correct or incorrect answer.

### IVR mLearning Processes

**FIGURE 1.**
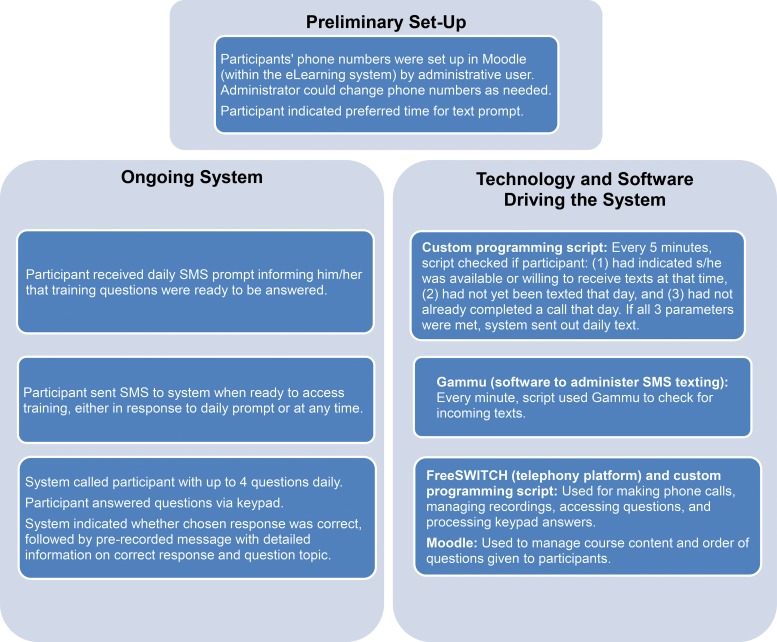
Overview of Interactive Voice Response mLearning Training System

We provided a 3-hour orientation for mLearning participants at the regional medical office in Thiès, Senegal. Figure 1 explains the IVR mLearning processes and the accompanying technology. We set up the participants’ personal mobile phone numbers in the mLearning system before starting the course. The system sent a daily prompt via SMS text to participants’ phones asking if they were available and ready to engage with the course content. When the participants were ready, even if it was hours later, they texted the mLearning system, and the system immediately called with the recorded voice questions and explanations. The participants selected the answer to each question using their telephone keypad and heard the detailed explanation whether they selected the correct or incorrect answer.

Participants texted the mLearning system when they were ready to engage with course content, and the system immediately called with the recorded voice questions and explanations.

Participants could opt to answer between 0 to 4 questions a day based on their preference and availability, and they could send SMS texts to the system and receive calls at any point during the day as long as they had not already answered 4 questions that day. This feature was included in case participants wanted to access the questions earlier than the predetermined prompt time, had issues with a call, had to hang up for other reasons, or wanted to answer additional questions. Participants received a text when they reached their daily limit of questions.

After participants answered all 20 questions once, they received the same questions and explanations a second time. Once the participants answered a question correctly twice, the question was retired and not asked again. Every day during the second round, participants received questions they answered incorrectly until they answered every question correctly twice, at which point the questions were retired. Participants successfully completed the course when all questions had been retired. After completion of the course, participants received a printed copy of the course content for future reference (see supplementary materials).

### IVR mLearning Training System Technologies

**FIGURE 2.**
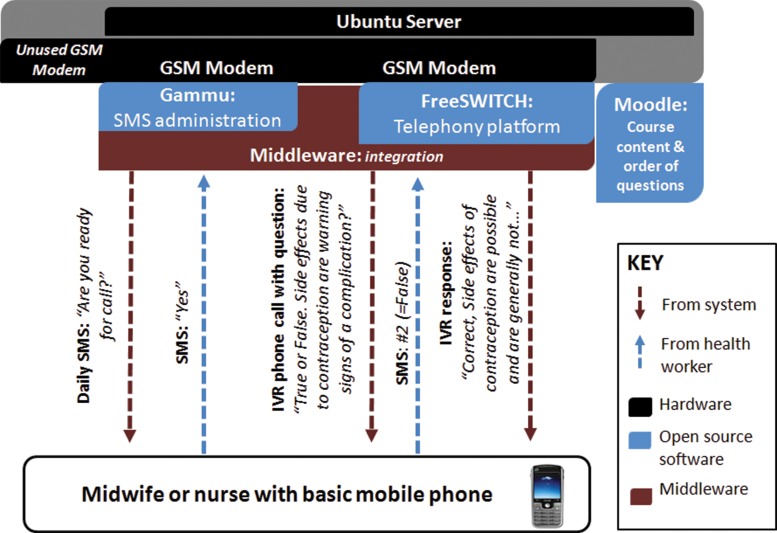
Interactive Voice Response mLearning System Infrastructure

Figure 2 shows the basic hardware and software infrastructure used in the IVR mLearning system. The system ran on an Ubuntu server and 2 GSM modems. We developed a set of custom scripts (“middleware”) to manage interactions between open source IVR software tools and learning solutions. FreeSWITCH—an open source telephony platform—handled the voice interactions via one modem and Gammu software interfaced with the other modem to administer the SMS texting. Moodle, the predominant open source eLearning system, managed the quiz interactions. We prioritized using open source technologies because of their low cost and ability for local adaptations.

The system logged all the texts that were sent and received and monitored what questions were asked and answered during each call on a daily basis. The project coordinator monitored the IVR mLearning system and contacted any participants who had not accessed the training system in over a week to determine if they were experiencing problems with the system. Identified problems were resolved as quickly as possible.

The project enrolled the IVR mLearning system and participants in a 2-month “friends & family” contract with a Senegalese telecommunications provider at a cost of approximately US$20 per participant per month. The contract allowed participants to send and receive text messages to the IVR mLearning system at no charge and the IVR system to make unlimited calls to participants.

## ASSESSMENT METHODS

### Design and Data Collection

To assess feasibility, we monitored implementation of the IVR training using administrative data from the mLearning system, such as the number of text messages sent and the duration and time of each IVR call. In addition, trained data collectors visited participants at their health posts within 5 weeks of course completion to administer a survey about participants’ opinions and experiences. Finally, we used a pre/post-intervention study design with no comparison group to assess changes in participant knowledge during the initial pilot. The written pre- and post-test consisted of 20 multiple choice and true/false questions. Participants completed the pre-test at orientation (February 2013) and the post-test both during the post-training survey (April 2013) and during a supervision visit 10 months after completing the IVR training course (February 2014). Participants did not receive any additional training, supervision, or mentoring in management of contraceptive side effects or misconceptions during the IVR training course or within 10 months after the course.

### Data Entry and Analysis

We calculated trends and patterns in participant and system activity using Microsoft Excel and Stata 13. The opinions and experiences of providers were expressed as proportions. We used McNemar’s exact chi-square statistic for paired binomial outcomes with small sample sizes^34^^,^^35^ to determine any significant changes in correct answers to individual knowledge questions between the pre- and post-test. To assess the significance of change in the overall knowledge scores (out of 20 questions) on the pre-test and post-test, we used the Wilcoxon signed rank test, a non-parametric test.^34^

### Research Ethics

The protocol was reviewed and approved by the national review board for research ethics in Senegal (*Comité National d’Ethique Pour la Recherche en Santé*). All participants provided voluntary and informed consent.

## RESULTS

### Participant Characteristics

The sample was almost evenly split between men and women (Table 1). Participants were midwives, nurses, nursing assistants, and health agents, the majority of whom (90%) worked in health posts. Most (65%) were from rural areas, and the majority (75%) reported provision of family planning services multiple times a day. All participants used their own mobile phone to access the course, and all had previously used their mobile phones for other work-related activities.

**TABLE 1.** Characteristics of mLearning Participants, Senegal 2013 (N = 20)

**Characteristic**	**No. (%)**
Diploma/post	
Midwife	7 (35)
Nurse	6 (30)
Nursing assistant	5 (25)
Health agent	2 (10)
Age group, years	
30–34	9 (45)
35–44	5 (25)
45–56	6 (30)
Sex	
Male	11 (55)
Female	9 (45)
Type of facility	
Health post	18 (90)
District hospital	2 (10)
Facility location	
Urban	7 (35)
Rural	13 (65)
Frequency of provision of family planning services^a^	
Multiple times a day	15 (75)
Once a day	3 (15)
Once a week	2 (10)
Contraceptive method most often requested by clients^a^	
Injectable contraceptives	17 (85)
Contraceptive pills	3 (15)
Personal mobile phone used for the course^b^	20 (100)
Previously used mobile phone for any work-related activity	20 (100)
Referred a patient	16 (80)
Requested stock	13 (65)
Received work-related information/guidance	11 (55)
Submitted data to the Ministry of Health or other stakeholders	7 (35)
Scheduled work hours	4 (20)
Training	2 (10)
Other	5 (25)

a As reported by provider.

b Four participants shared their phone with someone else, although only one reported that it made it difficult at times to complete the course.

### Feasibility of Implementation

All participants completed the course. The majority completed it within 5 weeks while 1 participant required 9 weeks. Administrative data indicate that throughout the course the system sent 620 prompt texts, and participating health workers texted the system a total of 640 times to prompt a call. The system made 619 calls using IVR, although only 496 (80%) of these calls resulted in administration of the spaced-education questions to participants.

The majority of participants completed the mobile training course within 5 weeks.

Some participants (30%) reported dropped IVR calls, often due to do poor network reception (Table 2).

30% of participants reported dropped IVR calls, often due to poor network reception.

**TABLE 2.** Participants’ Reported Experiences With Receipt of IVR mLearning System Calls, Senegal 2013 (N = 20)

**Experiences**	**No. (%)**
Received text message prompt from the system every day	15 (75)
Frequency of receiving IVR call after texting the system as ready	
Always (100% of the time)	13 (65)
Frequently (75% of the time)	5 (25)
Sometimes (50% of the time)	2 (10)
Average time to receiving IVR call after texting the system	
Less than 15 minutes	16 (80)
Between 15 and 30 minutes	4 (20)
More than 30 minutes	0 (0)
Frequency of dropped IVR calls	
Never	14 (70)
Infrequently (1–4 times)	4 (20)
Sometimes (5–9 times)	2 (10)
Ability to receive training questions	
Always able to receive	9 (45)
Infrequently unable to receive (1–4 times)^a^	8 (40)
Sometimes unable to receive (5–9 times)^a^	3 (15)
IVR voice recording was easy to understand	20 (100)

a Reasons for infrequently or sometimes not receiving training questions (number of respondents): poor reception (5); no call from system (4); issues with phone number, airtime, or phone charge (3); and inability to hear audio (1).

Three-quarters of participants reported receipt of a daily text prompt, and 90% always or frequently received a return IVR call after accepting the prompt (Table 2). According to administrative data, the system called participants, on average, within less than 10 seconds of receiving a participant’s prompt for call. During the first week, we experienced issues with the telephone network contract that affected system functioning, but these problems were quickly resolved. All participants reported the IVR audio recording was easy to understand.

**FIGURE 3.**
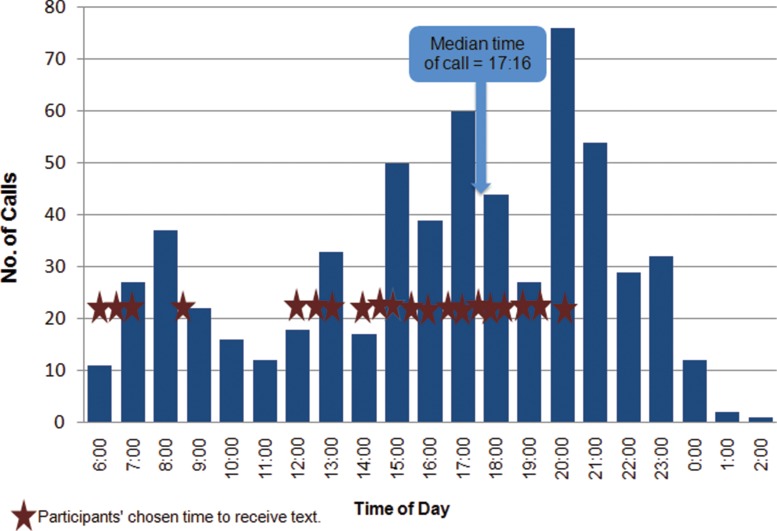
Timing of Interactive Voice Response Calls Throughout Duration of the mLearning Training Course, Senegal 2013 (N = 619 Calls)

The most common time of day for calls was in the late afternoon and evenings (median time, 5:16 pm) (Figure 3). Although all but 4 participants initially requested not to be contacted by the system after 8:00 pm, approximately one-third of the IVR calls were initiated by participants (through SMS text) after 8:00 pm. The majority of IVR calls were prompted and received outside normal working hours.

Most calls were made outside normal working hours.

On average, participants attempted to answer 2 questions per day. The average call time was 12 minutes and 52 seconds. One call in the second week of the course was logged as lasting 2 hours and 50 minutes; we excluded this from our analyses because we believe it may have been an error in the system.

### Acceptability

**FIGURE 4.**
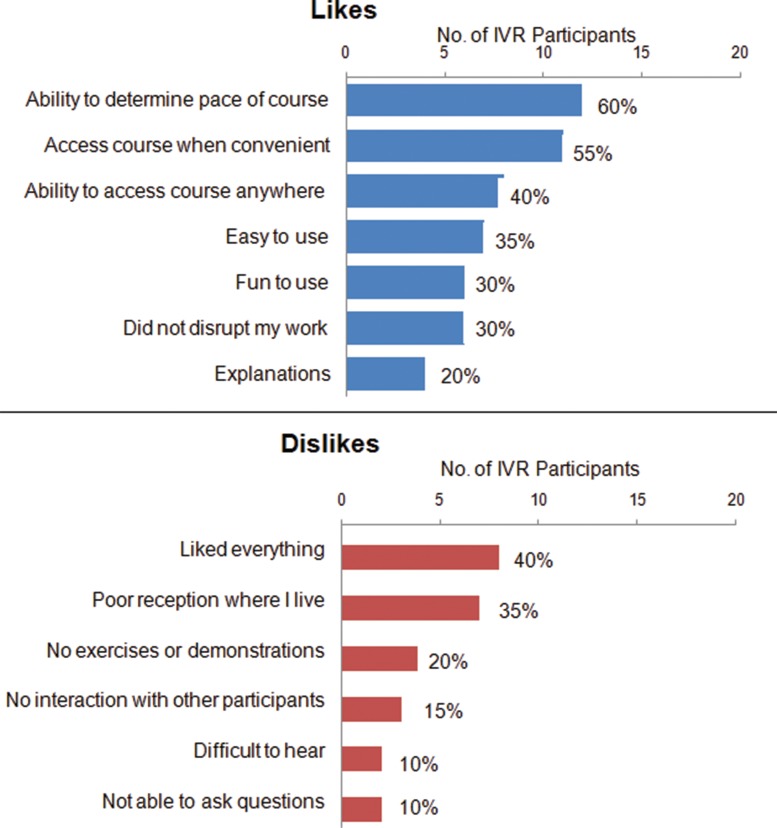
Participants’ Reported Likes and Dislikes^a^ About Using Mobile Phones in the mLearning Training Course, Senegal 2013 (N = 20) Abbreviation: IVR, interactive voice response. ^a^Participants could provide multiple responses for likes and dislikes. No participants responded that the mobile course was difficult to use or too long to complete.

**FIGURE 5.**
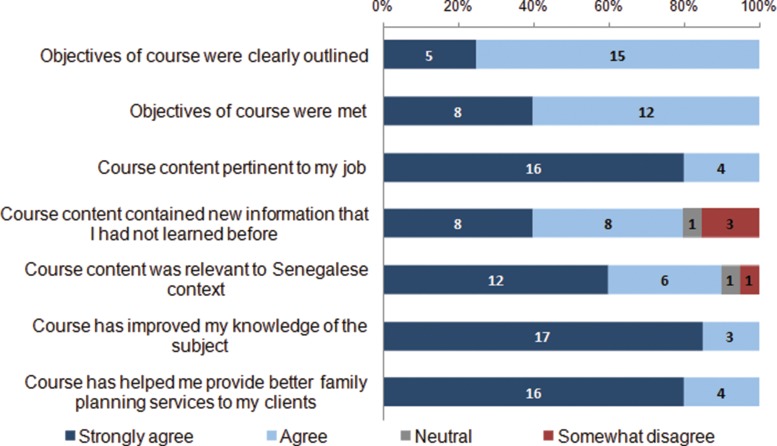
Participants’ Ratings of the mLearning Training Course Content, Senegal 2013 (N = 20)^a^ ^a^Number shown on bars represents the number of participants providing that rating. No participants “strongly disagreed” with any of these statements.

Participants reported very positive **experiences** with using their mobile phones for training (Table 3). The large majority (90%) noted that using the phone for the course was easy or very easy and that they learned the same or more compared with an in-person course. Participants expressed appreciation for the ability to determine the pace of the course (60%), convenience (55%), and flexibility to access the course anywhere (40%) (Figure 4). The largest criticism was poor mobile network reception (35%). Some participants also noted that the mobile medium did not allow for practical exercises or demonstrations (20%) or interaction with other participants (15%). Most participants agreed or strongly agreed that the course contained new information that they had not learned before (80%) and that the refresher training was pertinent to their jobs (80%) (Figure 5). A large majority strongly agreed that the course improved their knowledge on the subject (85%) and helped them provide better services to their clients (80%).

Participants liked that they could set their own pace and access the mobile course anywhere.

**TABLE 3.** Participants’ Opinions About Using Mobile Phones for Training, Senegal 2013 (N = 20)

****	**No. (%)**
Overall experience	
Very good	13 (65)
Good	7 (35)
Neutral/bad/very bad	0 (0)
Ease of using a mobile phone to complete training course	
Very easy	8 (40)
Easy	10 (50)
Difficult	2 (10)
Amount of learning on mobile phone vs. in-person course	
Learned more	12 (60)
Learned the same	6 (30)
Learned less	2 (10)
Would like to take another course on mobile phone	20 (100)

Table 4 presents participants’ **preferences for delivery of training** using the mLearning system. Most participants stated they preferred to receive prompt texts once a day (65%), the ability to answer up to 4 questions a day (65%), and the requirement of 2 correct responses before a question is retired (60%), which was how the course was delivered in this pilot application of the IVR mLearning system. Notably, 45% of participants stated that 20 questions in the course were too few, 45% thought the explanations provided too much information, and 55% noted that the 8-week duration for the course was too long. In an open-ended question for any recommendations, participants most often recommended the extension of the system to other health topics (8 recommendations) and the expansion of the family planning course to other health workers (7 recommendations).

**TABLE 4.** Participants’ Preferences for IVR mLearning System, Senegal 2013 (N = 20)

**Preferences**	**No. (%)**
Preferred frequency of prompt texts	
More than once a day	6 (30)
Once a day*	13 (65)
Every 2–3 days	1 (5)
20 questions included in the course was:	
Too many	1 (5)
Right amount	10 (50)
Too few	9 (45)
Preferred maximum number of questions to answer per day	
1–2	3 (15)
3–4*	13 (65)
5–9	4 (20)
Preferred number of times question must be answered correctly before being retired	
More than 2 times	6 (30)
Two times*	12 (60)
Only once	2 (10)
Amount of information in the explanations was:	
Too much	9 (45)
Right amount	11 (55)
Too little	0 (0)
Course duration of 8 weeks was:	
Too long	11 (55)
Right amount	9 (45)
Too short	0 (0)

* Asterisked items indicate the frequency or amount used in the pilot mLearning course.

### Provider Knowledge

The number of times participants answered the training questions correctly varied by topic (data not shown). For example, all participants correctly answered a question about the side effects of pills on both attempts, while incorrect answers were more common for side effects of IUDs and implants.

**FIGURE 6.**
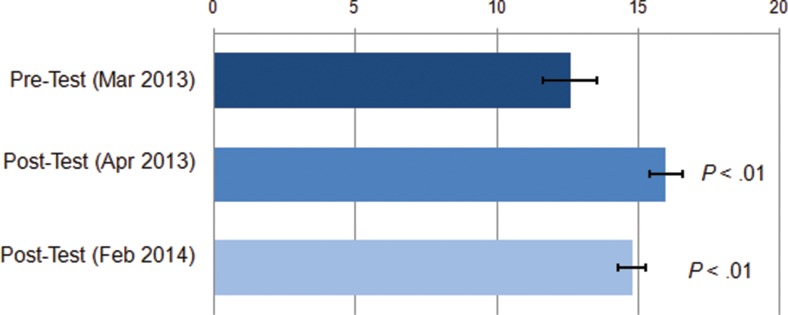
Average Number of Questions^a^ Answered Correctly Before (March 2013) and After (April 2013 and February 2014) the mLearning Training Course, Senegal 2013 (N=20) ^a^ Total of 20 questions related to contraceptive side effects and misconceptions. Statistical significance (*P* values) calculated using Wilcoxon signed-rank test comparing each post-test to the pre-test.

Overall, participants scored relatively well on the pre-test before the training, answering, on average, 12.6 questions about contraceptive side effects and misconceptions correctly out of 20 (Figure 6). After the training, the average score increased significantly to 16.0 correct questions. There was a slight decline in average knowledge scores 10 months after the post-test (to 14.8), but the knowledge level was still significantly higher than before the training. Between pre-test and the first post-test, significantly more participants answered correctly the questions on side effects of condoms, emergency contraceptive pills, and injectables, as well identifying misconceptions about LAM (results not displayed).

Knowledge scores increased significantly after the mobile training.

## DISCUSSION

An innovative mLearning system that delivers refresher training via simple mobile phones using IVR and SMS text was appropriate, feasible to implement, acceptable to health workers, and associated with gains in knowledge in the short-term and 10 months after the training. The IVR mLearning system using the spaced-education approach holds great promise to train health workers across health disciplines using simple mobile phones in areas that do not have good Internet connection. As with other mHealth initiatives, the reliability of cellular networks in remote areas remains a challenge.

mLearning systems using interactive voice response hold potential as a health worker training solution, particularly in low-resource settings without good Internet connection.

Participants prompted the majority of IVR calls during non-regular working hours and the average call time was about 13 minutes, suggesting that the IVR mLearning system training did not disrupt the health workers’ service delivery. In comparison, a conventional in-service training workshop would require trainees to leave their posts for a number of days. Participants in this mLearning pilot did have to leave their site of service for the half-day orientation. However, participants noted that the orientation prepared them well for the course, and previous experiences in mHealth initiatives have highlighted the importance of good orientation for participants.^16^

Participants prompted most IVR calls outside of normal working hours, suggesting little or no disruption to service delivery.

Participants highly appreciated the convenience of the system—the ability to determine when and where to access the training and the pace of completing the course. The IVR mLearning system included some interaction and question repetition—training characteristics that previous studies have found to be effective in self-directed learning, because participants can learn at their own pace and access the information when convenient.^1^

### Challenges and Considerations

One of the inherent purposes in our pilot deployment of the IVR mLearning system was to identify issues and challenges requiring improvements before any implementation on a larger scale.

#### Network Contracts

During the first week of implementation, we experienced challenges related to the contract with the telephone network and loading of credit on the mobile phones for airtime, issues similar to those found in other pilot studies of mHealth applications.^16^ We were able to resolve these issues relatively quickly, and a local coordinator facilitated resolution of other issues with the network carrier and participants over the duration of the course. However, future applications should allow sufficient time to test airtime and contract mechanisms to avoid any issues with the network carrier during course delivery.

#### Cellular Network Coverage

A challenge in our pilot study, as with other similar mHealth initiatives, was the variability of the cellular network. About one-third of participants experienced problems with the cellular network, although all were able to complete the course within the allotted 8 weeks except for 1 participant who took 9 weeks. Future applications might assess network coverage in advance and recommend that health workers with limited coverage at their posts access the mLearning system when traveling to areas with better network reception. Additionally, working with multiple network providers, particularly in private-public partnerships, might ensure more reliable cellular reception.

#### Adaptations to the Operating System

FreeSWITCH, the platform that handled the voice interactions, encountered some issues with accessing multiple voice lines at the same time. This issue may be resolved through incorporation of a server/modem combination to handle individual calls, with one system managing all the interfaces to determine the timing of calls. This set-up would also facilitate the expansion of the system with new telephone lines. In this pilot application, participants found using a text to prompt an IVR call feasible and convenient. Future adaptations of the system for less literate learners might allow participants to prompt an IVR call with a simple call to the system, similar to the Mobile Academy system in Bihar, India.^26^ In addition, during the pilot, 1 participant was not aware that he had not completed the course. Future implementation should incorporate a progress report feature to text participants their daily or weekly progression in the course, as well as a planned phone call from the course coordinator to supervise progress and provide participants the opportunity to ask questions.

#### Course Duration and Content

We found that it was feasible to deliver the spaced-education course within about 8 weeks. More than half of the participants noted that the overall course duration was too long, although other participants took the full 8 weeks to complete the course of 20 questions. Almost half of the participants would have also preferred to have more questions included in the course, especially if the overall course duration remained at 8 weeks. The spaced-education approach relies on delivering material over time to increase learning and retention of the content; thus, substantially shortening the course may not result in the same improvements in knowledge. Although the contraceptive side effects and misconceptions course may not require changes to the duration, the IVR mLearning system can and should be adapted for different content and audiences, with shorter or longer course duration and more or fewer questions depending on the training content and needs and preferences of the learners. Future implementation of the IVR mLearning system could compare learning outcomes when the same course is given over different periods of time.

#### IVR mLearning Within Broader Training Needs

Although our results are encouraging, refresher training through mobile phones should complement—not replace—different approaches within an in-service training strategy and broader health systems support for health workers. Training delivered on simple mobile phones does not permit trainees to interact with the instructor and other participants, perform clinical practice/simulation, or view didactic images such as diagrams, photos, and graphs. Some participants disliked the lack of exercises or demonstrations and the inability to ask questions and interact with other participants. A few participants also suggested more contact with the system and course coordinator, as well as inclusion of written reference materials. Provision of supplementary written materials and incorporation of clinical exercises and/or interactive methods are essential for teaching and learning certain topics, and simple mobile technology may not be appropriate for certain pedagogic objectives. However, hands-on training and use of images are not necessary for all topics. For example, updates on regulations and guidelines for family planning or other health services could be communicated to health workers via a mobile phone, thereby ensuring standardization and quality assurance in communication on the topic. The mLearning system could also be well suited to meet emerging needs, such as Ebola prevention and management, requiring rapid dissemination of information to diffusely distributed health workers.

mLearning could be well suited to meet emerging health training needs requiring rapid dissemination of information, such as Ebola prevention and management.

Refresher training through mobile phones should complement, not replace, other in-service training approaches.

#### Costs

The largest cost associated with this pilot application was the technical assistance required to develop and install the IVR mlearning system (about US$42,000 for only the direct cost estimates; no overhead included). Other substantial costs included: (1) developing and revising the training content (about US$12,000); (2) orientation for participants on how to use the system (about US$1,000); and (3) coordination with participants and troubleshooting the system during the 8 weeks of the course (about US$2,000). Each participant received approximately US$20 per month in mobile telephone airtime, which enrolled them in a “friends & family” contract, enabling them to text the system and to send and receive unlimited free calls from and to the system. At the time, this type of contract was the most economical option for a system requiring considerable airtime to deliver IVR calls.

We anticipate that costs could be reduced in future, larger-scale applications. The system and software has now been developed, and costs of curriculum development could be reduced by using the same content or adapting standard training modules or policies, procedures, and norms. Orientation could take place during routine meetings of health staff and would be unnecessary if participants took a second course using the same system. The cost of telephone air time must be reduced in larger-scale applications. Similar to Mobile Academy in India,^26^ a toll-free number could be established, rather than establishing contracts for all participants. Also, many countries are moving toward cellular networks that allow health professionals to communicate with each other for free,^36^ and the IVR mLearning system could be incorporated into these networks.

### Study Limitations

We deployed and assessed the IVR mLearning system among a very limited number of selected participants. Working with a small sample allowed us to pilot test the IVR mLearning system with limited financial resources and to make changes based on participant feedback before attempting to deliver training with the system at a much larger scale. However, the small sample size, lack of a control group, and lack of any measurement on changes in health worker practices preclude conclusions about the overall effectiveness of the training system. Any larger-scale implementation needs to be accompanied by a rigorous evaluation that includes a comparison group and examination of health worker practices and quality of health services as the primary outcomes, using a comprehensive framework to document and assess the in-service training.^37^ We purposively selected providers to receive the IVR training, potentially limiting the external validity of our findings, even within the Senegalese context. Participants worked in both urban and rural settings, and health workers in rural areas did report more issues with cellular reception during the IVR mLearning system pilot (data not shown).

## CONCLUSION

The application of an mLearning system using IVR, SMS text messages, and simple mobile phones to provide refresher training to health care workers is feasible, well-liked by participants, and associated with improved participant knowledge. The IVR mLearning platform using the spaced-education approach has the potential to be an effective and efficient approach to providing refresher training and/or updates on national guidelines, policies, and protocols in family planning and other health service areas, and it can conveniently deliver robust refresher training content to remote health care workers without requiring them to leave their posts. It could easily be adapted and adopted to reach health workers with low literacy, since it relies mostly only on voice and numeric interactions, rather than written materials. The system also has the potential to overcome language barriers, as training messages can be recorded in any language or dialect. The system should be scaled-up to other geographic areas and training topics in Senegal, with implementers giving sufficient attention and financial resources to the system architecture, organizational and administrative systems, as well as training and supervision for staff to manage the system.^38^ Close monitoring and rigorous evaluation of larger-scale implementation, including considerations of costs and sustainability, can provide robust evidence that the IVR mLearning system is feasible and effective at scale.

## Notes

**Peer Reviewed**
